# Clinical and Immunological Outcomes of HIV-Exposed Uninfected and HIV-Unexposed Uninfected Children in the First 24 Months of Life in Western Kenya

**DOI:** 10.21203/rs.3.rs-3073433/v1

**Published:** 2023-06-29

**Authors:** Jessica E. Ray, Katherine R. Dobbs, Sidney O. Ogolla, Ibrahim I. Daud, David Midem, Maxwel M. Omenda, Amy S. Nowacki, James G. Beeson, Katherine R. Sabourin, Rosemary Rochford, Arlene E. Dent

**Affiliations:** Case Western Reserve University; Case Western Reserve University; Kenya Medical Research Institute; Kenya Medical Research Institute; Kenya Medical Research Institute; Kenya Medical Research Institute; Cleveland Clinic Lerner Research Institute; Burnet Institute; University of Colorado; University of Colorado; Case Western Reserve University

**Keywords:** HIV-exposed uninfected, HEU, HIV, malaria, infant, child, growth, cytokine, vaccine, antimalarial antibodies

## Abstract

**Background:**

Previous studies show increased morbidity in children who are HIV-exposed but uninfected (HEU) compared to children who are HIV-unexposed uninfected (HUU). We sought to evaluate the effects of prenatal HIV exposure on clinical and immunological outcomes in the first 24 months of life.

**Methods:**

Eighty-five HEU and 168 HUU children from Kenya were followed from birth to 24 months. All mothers with HIV received combination antiretroviral therapy. HEU children received standard-of-care cotrimoxazole prophylaxis through 18 months. Episodes of acute illness were identified through a combination of active and passive follow up. Trajectories of plasma cytokines, vaccine-specific antibodies, and antimalarial antibodies were examined.

**Results:**

HEU and HUU children had similar growth curves. HEU children had lower rates of malaria and respiratory illness. Trajectories of plasma cytokines and vaccine-specific antibodies were similar in HEU and HUU children. There were subtle differences in antimalarial antibody dynamics, in which HEU children had overall lower antibody levels against five of the 14 malaria antigens tested.

**Conclusions:**

HEU children born to optimally treated mothers living with HIV had similar growth characteristics and immune profiles compared to HUU children. HEU children had reduced risk for malaria and respiratory illness, which may be secondary to cotrimoxazole prophylaxis.

## Background

HIV continues to be a major public health threat, especially in sub-Saharan Africa. Maternal HIV infection is a risk factor for adverse pregnancy and birth outcomes for millions of maternal-neonatal dyads, including maternal and fetal anemia, preterm birth, and low birth weight (1). Antiretroviral therapy (ART) during pregnancy prevents mother-to-child transmission of HIV and improves maternal health (2–5). Currently, most HIV-infected pregnant women have access to ART (6). In sub-Saharan Africa, 69% of HIV-infected pregnant women had access to ART in 2019 (7). With increased access to ART there is a growing population of HIV-exposed but uninfected (HEU) infants. The estimated number of HEU infants reached 14.8 million in 2018, and of these 13.2 million are from sub-Saharan Africa (8).

HEU infants are healthier than HIV-infected infants (9), but several studies have shown that HEU infants have greater morbidity and mortality than HIV-unexposed uninfected infants (HUU) (10). HEU infants have been shown to have a greater frequency of all-cause sick clinic visits (11), greater infection rates (12), and a 1.2–2.7 times greater risk of hospitalization (13, 14) compared with HUU infants. Mortality rates for HEU infants have been shown to be 3–4 times greater than in HUU infants (9, 15). The underlying mechanisms for these observations are likely multifactorial and may be influenced by parental illness and death, increased infectious exposures, impaired placental transfer of protective maternal antibodies, antiretroviral therapy exposure *in utero*, and subsequent immune modulations.

Many of these conclusions were drawn from studies performed prior to the widespread implementation of optimal HIV therapies during pregnancy. We have previously examined the immunological consequences of maternal HIV infection among a maternal–neonate cohort of HIV + women on ART and HIV-women living in a malaria-endemic area of western Kenya (16). Our results showed that even in optimally treated HIV + women, defects in transplacental transfer of antimalarial antibodies persisted, though we found no effect of maternal HIV infection on birth outcomes, cord blood plasma cytokine profiles, or transplacental transfer of vaccine-specific antibodies. Here, we report on 24 months of follow up for HEU and HUU children in this cohort with investigations of growth trajectories, rates of malaria and other common childhood illnesses, and trajectories of plasma cytokine profiles, vaccine-specific antibodies, and antimalarial antibodies.

## Methods

### Ethical Approval

Informed consent was obtained in the appropriate local language. Ethical approval was obtained from the University Hospitals Cleveland Medical Center IRB (8–7–9), Colorado Multiple IRB (15–1277), and Kenya Medical Research Institute (KEMRI) Scientific and Ethical Review Unit (NON SSC 089).

### Study Site and Participants

The study was conducted in Kisumu County, Kenya, at the Chulaimbo Sub-County Hospital, from 2011–2016. Malaria transmission in this area is intense and year-round, with peaks coinciding with rainy seasons (17). Chulaimbo Hospital serves a primarily rural population and is an Academic Model Providing Access to Healthcare (AMPATH) site. Clinical services and medications for HIV + patients and their families are supported by USAID and the Indiana University–Kenya partnership.

Participants were enrolled in a prospective observational study in which HIV + and HIV-pregnant women were enrolled at their first prenatal visit (typically during the second trimester) and followed through pregnancy. Infants were followed from birth to 24 months of age. All HIV + women received ART therapy (Lamivudine + Zidovudine (Hb > 8g/dL) or Tenofovir (Hb < 8g/dL) + Nevirapine (CD4 < 250 cells/μL) or Lopinavir/ritonavir (CD4 > 250 cells/μL)). HEU newborns were treated with one dose of Nevirapine after delivery and 6 weeks of Zidovudine, with zero HIV + infants in this cohort.

Eighty-five HEU and 168 HUU children with complete clinical data and samples were included in this study. Study visits occurred at birth and at 6, 10, 14, and 18 weeks and 6, 9, 12, 15, 18, 21, and 24 months of age. The Kenya Expanded Programme on Immunization schedule included diphtheria-pertussis-tetanus (DPT), *Haemophilus influenzae* type b (Hib), hepatitis B, oral polio (OPV) and pneumococcus (PCV) vaccines at 6, 10, and 14 weeks and measles vaccine at 9 and 18 months of age. According to Kenyan Ministry of Health guidelines, HEU infants received cotrimoxazole prophylaxis from 6 weeks of age until cessation of breastfeeding and definitive exclusion of HIV infection (typically at 18 months of age).

### Clinical Events

Data collected during each visit included clinical history, physical exam and anthropometric measurements, and any concurrent diagnoses (e.g., upper respiratory tract infection, malaria, gastroenteritis). Participants were seen at the study clinic for any interim sick visits, where data were collected regarding physical exam findings, diagnoses, treatments given, and severity of illness (any illness that required hospitalization was classified as severe). Major diagnostic categories from interim or concurrent sick visits included clinical malaria (defined as febrile illness with *Plasmodium falciparum* (Pf) parasitemia by blood smear or rapid diagnostic test), upper respiratory tract infection (URTI), any respiratory illness, gastroenteritis/diarrhea, and any severe illness.

### Blood Sample Collection and Processing

At delivery, venous cord blood samples were collected in heparinized syringes. At all other visits, heparinized finger prick or venous blood samples were obtained. Aliquots of 200 μL whole blood were stored at − 20°C. Plasma was separated and stored at − 80°C. All sample processing occurred within 1–5 hours of collection at the laboratory facilities at the Center for Global Health Research of KEMRI. All assays were conducted at the KEMRI laboratories.

### Detection of Pf infection by PCR

To determine the prevalence of asymptomatic Pf infection in the cohort, Pf PCR was performed on all available samples at 6, 9, 12, 15, 18, 21, and 24 month visits. DNA was extracted from whole blood using Qiagen QIAmp DNA Mini Kits. Pf PCR was performed as previously described (18).

### Measurement of Cytokines

Immunological assays were performed for a subset of children with plasma samples available at all timepoints. We measured levels of 12 cytokines in plasma samples from 59 HEU and 58 HUU children at birth, 6, 10, 14, 18, 26, 39, and 52 weeks of age. All plasma samples were assayed immediately after initial thawing. A multiplexed bead-based immunoassay was used to measure plasma concentrations of IFN-γ, IL-1β, IL-6, IL-10, IL-12P70, IL-17A, IL-17E, IL-17F, IL-21, IL-22, IL-23, and TNF (Human Th17 Magnetic Bead Panel, EMD Millipore).

### Vaccine-Specific IgG

We measured IgG antibodies to diphtheria, tetanus, hepatitis B, and measles in plasma from 61 HEU and 54 HUU children by ELISA, as previously described (16, 19, 20). Time points included birth, 6, 10, 14, and 18 weeks, and 6, 9, 12, 15, 18, 21, and 24 months. Serial dilutions of samples were compared with 5-point standard curves made with serial dilutions from World Health Organization–approved antigen-specific reference sera; diphtheria Ig, human (NIBSC 10/262, 2 IU/mL), tetanus Ig, human (NIBSC TE-3, 120 IU/ mL), hepatitis B Ig, human (NIBSC 07/164, 100 IU/mL), and measles Ig, human (NIBSC 97/648, 3 IU/mL).

### Pf Antigen-Specific IgG

We measured IgG antibodies to 14 recombinant Pf proteins in plasma samples from 69 HEU and 76 HUU children using Luminex MagPix assays (MagPlex, Luminex). Time points included birth, 6, 10, 14, and 18 weeks, and 6, 9, 12, 15, 18, 21, and 24 months. The assays were performed as previously described (16, 21). Supplementary Table 1 contains sources and quantities of conjugated antigens. For each assay, plasma was diluted 1:100 and 1:1000. The secondary antibody was R-Phycoerythrin-conjugated AffiniPure F(ab’) Fragment Goat Anti-Human IgG Fcγ Fragment Specific (Jackson ImmunoReaserch, West Grove, PA). Seven malaria-naïve North American adult plasma samples were tested on all plates as negative controls. Mean fluorescent intensity (MFI) values were divided by average MFIs of negative controls. Data are expressed as the fold-increase of the sample MFI relative to the negative control MFI (reported as fold over North American), as previously described (21).

### Statistical Analysis

World Health Organization (WHO) child growth standards were used to generate Z scores for weight-for-age (WAZ), length-for-age (LAZ), head circumference for age (HAZ), and body mass index (BMI) for age (BAZ) (WHO Anthro Survey Analyser, R package “anthro” v0.9.4) (22). Bayesian hierarchical regression analysis was used to fit WAZ, LAZ, HAZ, and BAZ linear growth curve models for the HEU vs. HUU groups, which accounts for both individual-specific effects and group-level effects. All models were fit using JAGS software via the R package “rjags” (23, 24). Missingness in the data was assumed to be completely at random. Prior distributions for group means for intercept and slope were weakly informative and specified as normal distributions with mean 0 and variance 100. Standard non-informative uniform distributions were specified for the residual variance and the standard deviations of intercept and slope. Posterior distributions were checked for convergence graphically and numerically using the $\hat{R}$ statistic; convergence criteria with $\hat{R}<1.1$ were met for all parameters for all models. For each growth curve model, posterior means for intercepts and slopes, with 95% highest probability density interval (HDI), were contrasted between HEU vs. HUU groups. We selected a region of practical equivalence (ROPE) with upper and lower limits of −0.05 and 0.05; contrast terms for group intercepts and slopes were deemed to be meaningfully different if the 95% HDI excluded this interval.

The prevalence of asymptomatic Pf infection among HEU and HUU infants was calculated at 6, 9, 12, 15, 18, 21, and 24 months of age. The relative risk of Pf infection in the HEU group compared to the HUU group was determined at each time point.

Clinical event rates for the major diagnostic categories (malaria, URTI, any respiratory illness, gastroenteritis/diarrhea, and severe illness) were calculated by the number of diagnoses per 1,000 person-months of follow up in each group. Rate ratios were calculated as the ratio of clinical event rates in HEU children divided by the clinical event rates in HUU children.

Time fixed effects regression models were used to analyze plasma cytokines, vaccine antibodies, and antimalarial antibodies in HEU vs. HUU children. We first tested whether trajectories differed among HEU vs. HUU children (“group x time” interaction effects). Then if the trajectories did not differ, we tested whether values for HEU children differed from values for HUU children (“group” main effect) and whether the values changed over time within a group (“time” main effect).

## Results

### Similar growth curves in HEU and HUU children

**Figure 1:**
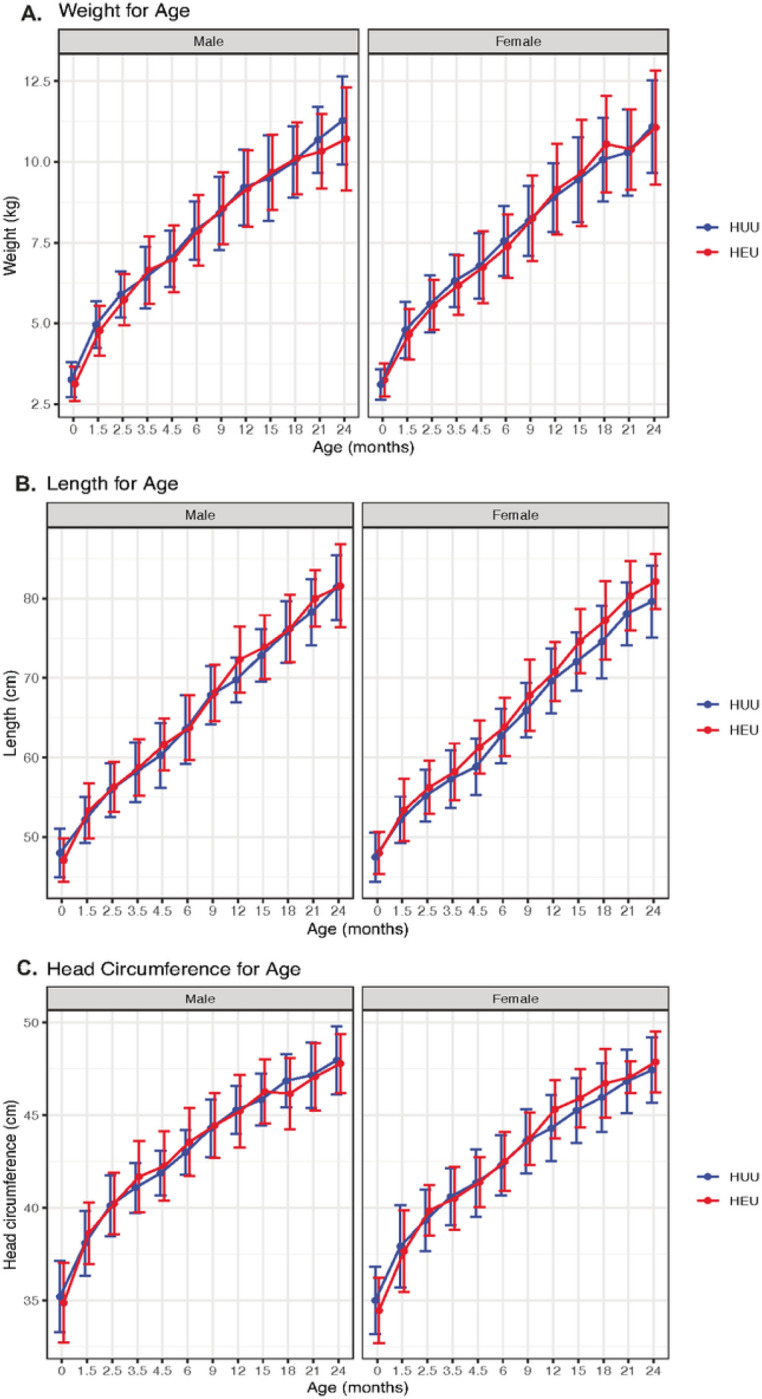
Similar growth curves in HEU and HUU children. Growth curves for **(A)** weight, **(B)** length, and **(C)** head circumference for age were assessed at 12 visits between birth and 24 months of age in male and female HUU (blue) and HEU (red) children. Mean and standard deviation at each time point plotted on the y-axis.

Birth characteristics for the cohort of 85 HEU and 168 HUU children are listed in Supplementary Table 2. There were no differences in gestational age at delivery, birth weight, length, or head circumference between HEU and HUU children. Mean gestational age was 37.2 weeks in HEU and 38.2 3.8 weeks in HUU children. Mean birth weight was 3.2 0.5 kg in both groups. The proportion of low birth weight was 8.4% in HEU children and 6.1% in HUU children (Chi-squared test p = 0.7). Growth curve trajectories for weight, length, and head circumference were similar among HEU and HUU children from birth to 24 months of age (Fig. 1). Linear growth curve models for Z scores for weight, length, head circumference, and BMI for age obtained using Bayesian hierarchical regression showed no statistically meaningful differences in HEU vs. HUU children (contrast terms for intercepts and slopes comparing each of the four growth curves for HEU vs. HUU were not credibly different from zero) (Supplementary Table 3).

### Lower rates of P. falciparum infection and respiratory illness in HEU vs. HUU children

**Figure 2:**
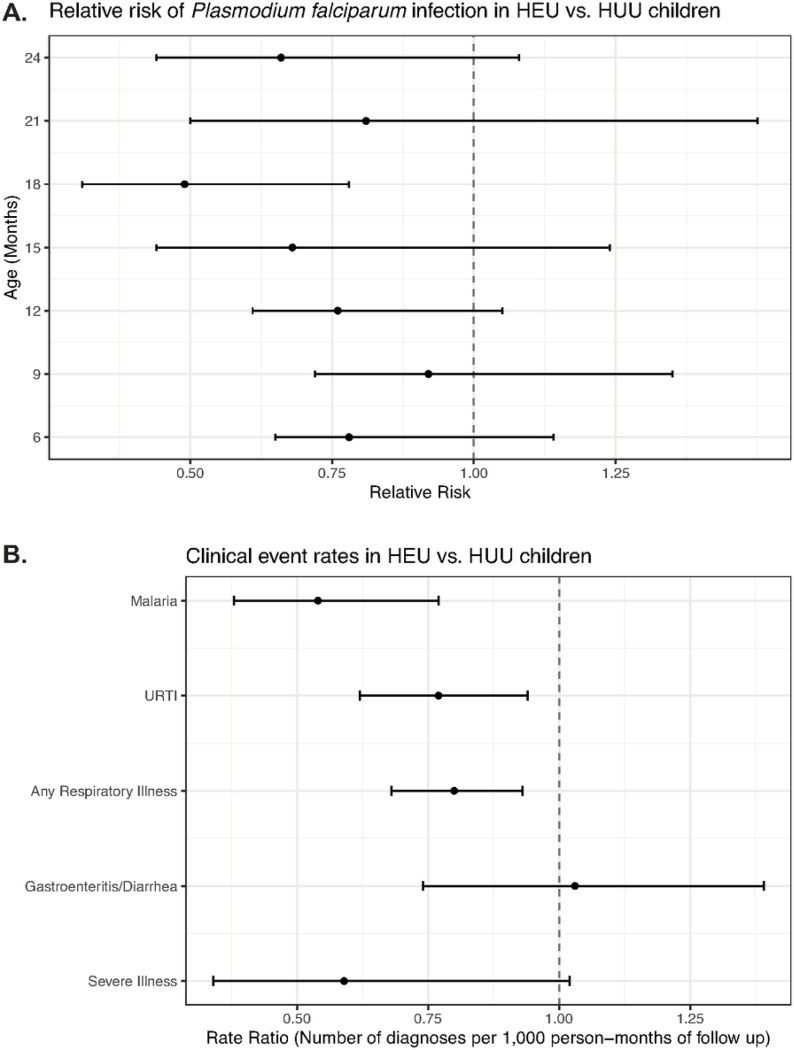
Rates of *P. falciparum*infection and clinical event rates in HEU and HUU children. **(A)** Relative risk of *P. falciparum* infection in HEU vs. HUU children between 6 and 24 months of age. P. falciparum infection detected by PCR. Relative risk less than one represents decreased risk in HEU children (point estimates and 95% confidence intervals displayed). **(B)** Differences in rates of clinical events in HEU vs. HUU children between birth and 24 months of age. Rate ratio less than one represents decreased rate in HEU children (point estimates and 95% confidence intervals displayed). URTI: upper respiratory tract infection.

The prevalence of asymptomatic Pf infection was measured at seven timepoints between 6 and 24 months of age in HEU and HUU groups. HEU children had reduced relative risk of Pf infection compared to HUU children at 18 months of age (RR = 0.49, 95% CI 0.31, 0.78) (Fig. 2A). There were no statistically significant differences in relative risk for Pf infection at the other timepoints.

Clinical event rates, including at scheduled follow-up visits and interim sick visits, were compared between HEU and HUU children between birth and 24 months of age (Fig. 2B). HEU children had lower rate ratios for malaria (0.54, 95% CI 0.38, 0.77), URTI (0.77, 95% CI 0.62, 0.94), and any respiratory illness (0.80, 95% CI 0.68, 0.93). There was no difference in gastroenteritis/diarrhea between the groups. The rate ratio for severe illness requiring hospitalization was lower in HEU children vs. HUU children, though this difference did not reach statistical significance (rate ratio = 0.59, 95% CI 0.34, 1.02).

### Similar plasma cytokine trajectories in HEU and HUU children

**Figure 3:**
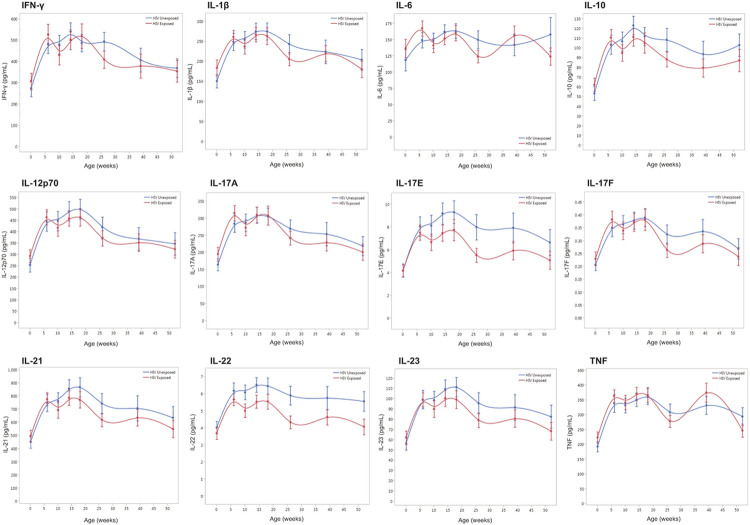
Twelve plasma cytokine trajectories in HEU and HUU children. Cytokines include IFN-γ, IL-1β, IL-6, IL-10, IL-12p70, IL-17A, IL-17E, IL-17F, IL-21, IL-22, IL-23, and TNF. HUU (blue) compared to HEU (red). Mean cytokine values plotted on the y-axis with error bars representing one standard error from the mean. Age (weeks) between birth and one year represented on the x-axis.

Trajectories for plasma levels of 12 cytokines were compared for 59 HEU vs. 58 HUU children at eight timepoints between birth and 52 weeks of age using fixed effects regression models (Fig. 3). There were no significant interaction effects between group and time for any of the 12 cytokines measured, indicating that the trajectories of these plasma cytokines did not differ between HEU and HUU children (Supplementary Table 4). We then evaluated the main effects for group for each of the cytokines. There was evidence for lower IL-22 levels in HEU children (mean 4.9 ng/mL) compared to HUU children (mean 5.7 ng/mL) (main effects p = 0.01). There was no evidence that plasma levels of the other 11 cytokines differed between HEU and HUU children. Finally, we evaluated the main effects for time to determine whether plasma cytokine levels changed over time independent of group and whether there were significant differences in cytokine levels at birth vs. 26 weeks (6 months) of age. For all 12 cytokines tested, the values did change over time. Specifically, 11 out of 12 cytokines were significantly higher at 26 weeks compared to birth, with the exception of IL-6 which showed no significant difference (Supplementary Table 4).

### Similar vaccine antibody trajectories in HEU and HUU children

**Figure 4:**
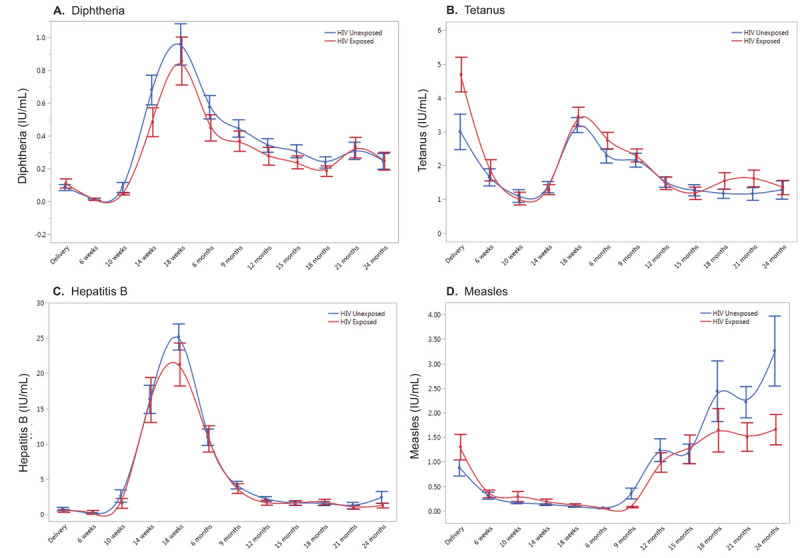
Trajectories of vaccine specific antibody levels in HEU and HUU children. Vaccine-specific antibody levels were measured for **(A)**Diphtheria, **(B)** Tetanus, **(C)** Hepatitis B, and **(D)** Measles vaccines. HUU (blue) compared to HEU (red). Age (birth to 24 months) is on the x-axis and vaccine specific antibody levels (IU/mL) are on the y-axis (mean with error bars representing one standard error from the mean).

Total IgG antibody responses to vaccines for diphtheria, tetanus, hepatitis B, and measles were compared between 61 HEU and 54 HUU children. Antibody concentrations were measured at 12 timepoints between birth and 24 months of age (Fig. 4). Fixed effects regression models were used to compare vaccine antibody trajectories between HEU and HUU children (Supplementary Table 5). There were no significant interaction effects between group and time for diphtheria, hepatitis B, and tetanus specific antibodies, indicating that the trajectories of these antibodies did not differ between HEU and HUU children. There was a significant interaction effect between group and time for measles-specific antibodies (p = 0.03). Separation between HEU and HUU trajectories appeared to occur beginning at 18 months of age (around the time that the second dose of measles vaccine is given), with higher anti-measles antibodies in HUU children (Fig. 4A). These data suggest that HUU children may have a heightened antibody response to the second measles vaccine dose compared to HEU children, though both groups of children generated antibody responses to the vaccine at levels associated with protection (25).

Because the separation between groups for anti-measles antibodies appeared to occur at the very end of the observation period, we proceeded with an additive model for all four vaccines. We evaluated the main effects for group which showed insufficient evidence that HEU and HUU children differed with respect to overall levels of any of the vaccine antibodies tested (Supplementary Table 5). Finally, we evaluated the main effects for time to determine whether vaccine specific antibody levels changed over time independent of group and whether there were significant differences in antibody levels at birth, 6 months, and 18 months of age. All four vaccine-specific antibody levels changed significantly over time, with rises in antibody levels coinciding with time of vaccination (18 weeks for diphtheria, tetanus and hepatitis B, and 9 months for measles) (Fig. 4, Supplementary Table 5).

### Differences in antimalarial antibody trajectories in HEU vs. HUU children may reflect differences in P. falciparum infection rates

**Figure 5:**
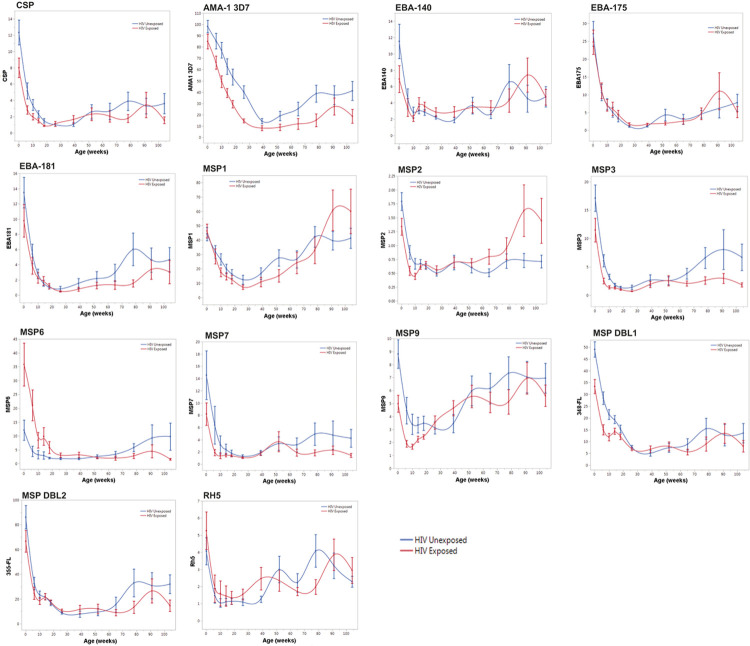
Fourteen anti-malarial antibody trajectories in HEU and HUU children. Malarial antigens include CSP, AMA-1 3D7, EBA-140, EBA-175, EBA-181, MSP1, MSP2, MSP3, MSP6, MSP7, MSP9, MSP DBL1, MSP DBL2, and RH5. HUU (blue) compared to HEU (red). Mean antibody values (fold over North American controls) plotted on the y-axis with error bars representing one standard error from the mean. Age (weeks) between birth and two years is plotted on the x-axis.

Antimalarial IgG antibodies to 14 Pf antigens were compared between 76 HEU and 69 HUU children. Antibody concentrations were measured at 12 timepoints between birth and 24 months of age (Fig. 5). Fixed effects regression models were used to compare antimalarial antibody trajectories between HEU and HUU children (Supplementary Table 6). There were no significant interaction effects between group and time for ten of the 14 antigens (CSP, EBA-140, EBA-175, EBA-181, MSP1, MSP3, MSP7, MSP9, MSP DBL2, and Rh5). Significant interaction effects between group and time were observed for four of the 14 antigens: AMA1–3D7 (p = 0.001), MSP2 (p = 0.01), MSP6 (p = 0.0002), and MSP DBL1 (p = 0.02) (Supplementary Table 6). There were no consistent patterns for differences in HEU vs. HUU children in trajectories of antibodies to these four antigens. Anti-MSP DBL1 antibody levels were slightly lower for HEU vs. HUU children at 18 and 24 months; anti-AMA1-3D7 antibodies were slightly lower for HEU children across all timepoints (Fig. 5). Given the slight differences between HEU and HUU children in trajectories for anti-AMA1-3D7 and MSP DBL1 antibodies, and the high degree of variability in the data, we proceeded with an additive model for these two antigens. The effect of HIV exposure on anti-MSP2 and anti-MSP6 antibody levels depended on age, with opposite correlations observed for each antigen. HEU children had lower anti-MSP2 antibodies compared to HUU children prior to 6 months of age, but after 6 months of age, HEU children had higher levels. Conversely, HEU children had higher anti-MSP6 antibodies compared to HUU children prior to 6 months of age, but after 6 months of age, HEU children had lower levels. (Fig. 5). We concluded that trajectories for anti-MSP2 and anti-MSP6 antibodies significantly differed between HEU and HUU children. Therefore, for these two antigens, we did not proceed to test main effects for group (to determine whether overall values differed in HEU vs. HUU) or main effects for time (to determine whether antibody values changed over time independent of group).

We evaluated main effects for group for 12 of the 14 antigens and found that HEU and HUU children significantly differed with respect to five of the 12 antigens included in the analysis: CSP, AMA1–3D7, MSP3, MSP9, and MSP DBL1. For all five of these antigens, HEU children had lower antibody levels than HUU children (Fig. 5, Supplementary Table 6).

We then evaluated main effects for time for the same 12 antigens, testing whether antibody levels changed over time in all subjects independent of group and whether there were significant differences in mean antibody levels at birth, 26 weeks (6 months), and 78 weeks (18 months) of age. For all twelve antibodies, values significantly changed over time (p < 0.0001) (Supplementary Table 7). Mean antibody levels were significantly lower at 6 months of age compared to birth for all twelve antigens included in this analysis (p < 0.0001). All mean antibody levels were higher at 18 months compared to 6 months, but the difference was statistically significant for only nine of the twelve antibodies tested (Supplementary Table 7).

## Discussion

Results from this cohort study in western Kenya indicate that HEU children born to mothers receiving ART had similar growth characteristics and immune profiles compared to HUU children. Notably, HEU children in this cohort had reduced risk for malaria and respiratory illness in the first 24 months of life, presumably secondary to standard of care for HEU children in this high malaria transmission area that includes cotrimoxazole prophylaxis from 6 weeks of age until cessation of breastfeeding and exclusion of HIV infection (~18 months of age).

Several studies conducted in sub-Saharan Africa demonstrate impaired weight gain and growth in HEU children compared to HUU children (26–29). The widespread implementation of lifelong ART for all HIV+ pregnant women (“Option B+”) may have a positive impact on infant and child growth. A study in Malawi compared growth in HEU children before and after Option B+ was implemented and found that postnatal weight gain was faster in HEU children born during the Option B+ period (30). A large prospective study of HEU and HUU children in South Africa in the context of Option B+ with breastfeeding showed that HEU children had small deficits in growth trajectories in the first 12 months of life, though overweight was common in both HEU and HUU infants at 12 months (31). A study in Rwanda showed considerable heterogeneity in growth patterns of both HEU and HUU children, in which birth characteristics such as infant sex, birth weight, and maternal height were predictive of growth trajectories in both groups (32). In this study, we did not detect a significant difference in growth trajectories between HEU and HUU children. Ongoing surveillance and research are needed to fully assess the impact of maternal HIV and ART exposure on child growth and metabolism in the context of universal maternal ART.

Numerous studies over the past several decades have shown increased infection-related morbidity and mortality in HEU children compared to HUU children (10, 12–15, 33–35). In the current study, we observed an opposite trend in which HEU children had fewer episodes of malaria and respiratory illness compared to HUU children. As with child growth, universal maternal ART likely has a positive impact on infectious morbidity in HEU children. Infants born to mothers without virological control during pregnancy have high rates of undernutrition and serious infectious morbidity (36), and pre-conception ART is associated with reduced risk for infection-related hospitalization (37).

Our cohort study took place in an area with intense year-round transmission of malaria. We suspect that in the context of universal maternal ART and breastfeeding, anti-malarial activity of cotrimoxazole underlies the decreased rates of *P. falciparum* infection and clinical malaria in HEU vs. HUU children (38, 39). While the WHO continues to recommend cotrimoxazole prophylaxis for all infants who are HEU (40), recent randomized trials observed no benefit of cotrimoxazole prophylaxis for HEU infants living in non-malarial areas of Botswana (41) and South Africa (42). Evidence from these trials, along with dramatic improvements in access to ART and healthcare for mothers and infants over the past 20 years, suggest that routine cotrimoxazole prophylaxis is not beneficial for all infants who are HEU (43). However, potential benefits of cotrimoxazole prophylaxis observed in this study and in other malaria hyper-endemic settings suggests the need to further develop perennial malaria chemoprevention strategies targeting all children independent of HIV exposure.

We did not observe significant differences in plasma cytokine trajectories over the first year of life. In the same cohort, we observed lower levels of peripheral blood plasma cytokines in HIV+ vs. HIV-mothers at delivery, though we did not see differences in cord blood plasma cytokine levels in HEU vs. HUU neonates (16). As with clinical outcomes of child growth and infection-related morbidity, maternal access to ART in pregnancy likely plays a significant role in fetal and infant immune development. A recent study examined innate immune cytokine responses in maternal peripheral blood, placental blood, and neonatal cord blood and found that the effects of HIV on maternal and infant innate immunity were restricted to women who did not receive ART before pregnancy (44). Multifactorial influences on infant immune development were highlighted in another recent study that examined immune and microbiome differences in HEU vs. HUU 2-year-olds across diverse geographical settings (45). The study revealed immune differences between HEU and HUU children that were site-specific: differences in innate immune responses distinguished HEU and HUU children in Belgium and Canada, though no immune differences were noted between South African HEU and HUU children (45). These results suggest that, depending on the setting, other factors may influence immune phenotypes such that any effect of HIV or ART exposure is no longer evident.

In this study, longitudinal antibody responses to vaccination against diphtheria, hepatitis B, tetanus, and measles were similar in HEU and HUU children. Our results did suggest that HUU children may have a heightened response to the second dose of measles vaccine compared to HEU children, though both groups generated levels of anti-measles antibodies associated with clinical protection (25). This is in line with prior studies showing that HEU children generally mount robust antibody responses to vaccines, including pneumococcal conjugate, tetanus, pertussis, hepatitis B, and Haemophilus influenzae type B (46–49). We did observe differences between HEU and HUU children in overall levels of antimalarial antibodies against five of 14 Pf antigens studied, in which antibody levels were lower in HEU children. We suspect this may reflect differences in rates of Pf infection and clinical malaria in HEU children, though more research is needed to confirm these findings and to determine whether these differences have any impact on long-term acquisition of clinical immunity to malaria.

## Conclusions

HEU children born to mothers with HIV on ART had similar growth characteristics and immune profiles compared to HUU children. In this malaria hyper-endemic setting, HEU children had decreased risk for malaria and respiratory illness, which may be secondary to cotrimoxazole prophylaxis. These results highlight the importance of universal access to ART and high-quality healthcare for women with HIV to optimize outcomes for both mothers and children.

## Availability of data and materials.

The datasets used and analyzed during the current study are available from the corresponding author on reasonable request.
